# Global Adoption of Value-Based Health Care Initiatives Within Health Systems: A Scoping Review

**DOI:** 10.1001/jamahealthforum.2025.0746

**Published:** 2025-05-16

**Authors:** Ayooluwa O. Douglas, Senthujan Senkaiahliyan, Caroline A. Bulstra, Carol Mita, Che L. Reddy, Rifat Atun

**Affiliations:** 1 Health Systems Innovation Lab, Harvard T.H. Chan School of Public Health, Harvard University, Boston, Massachusetts; 2 Department of Global Health and Population, Harvard T.H. Chan School of Public Health, Harvard University, Boston, Massachusetts; 3 Countway Library, Harvard University, Boston, Massachusetts; 4 Institute of Health Policy Management and Evaluation, University of Toronto, Toronto, Ontario, Canada; 5 Peter Munk Cardiac Centre, Toronto General Hospital, University Health Network, Toronto, Ontario, Canada

## Abstract

**Importance:**

Health systems worldwide are facing several contextual challenges threatening their sustainability, including aging populations with complex health care needs, workforce shortages, and persistent health disparities, which are driving health care costs. Optimizing health systems to respond to contextual challenges and offer quality care for all requires innovative frameworks like value-based health care (VBHC) and high-value health systems (HVHS) frameworks that focus on improving patient outcomes while minimizing costs.

**Objective:**

To examine how value-based initiatives have been introduced in health systems worldwide.

**Evidence Review:**

A comprehensive literature search was conducted across MEDLINE/PubMed, Embase, Health Business Elite, and Web of Science Core Collection. The search included controlled vocabulary terms relevant to VBHC and covered publications between January 1, 2007, and July 7, 2023. After title and abstract screening, followed by full-text review, experimental, observational, and case studies that examined the implementation of the VBHC framework or its elements were included. Articles that focused solely on insurance, cost-effectiveness analysis, theoretical models without implementation, nonempirical studies (eg, reviews, commentaries), and gray literature (eg, news articles) were excluded.

**Findings:**

Of 11 948 articles initially identified for potential inclusion, the final sample included 50 initiatives, with 47 from high-income countries, 2 from upper-middle–income countries, and 1 from a lower-middle–income country. The review revealed that VBHC adoption remains confined to the departmental or institutional level, with few examples of systemwide or national implementation. Although many initiatives integrated various elements of the VBHC framework and components of the HVHS model, none achieved full implementation of all aspects.

**Conclusions and Relevance:**

This scoping review showed that since its formal introduction in 2006, VBHC has been widely recognized as a strategy for improving health system performance, but large-scale adoption will require a strategic shift toward integrating value-based components at national and regional levels. These findings highlight the need for research on effective implementation models, particularly in lower-resource settings, to guide policymakers and health system leaders in scaling VBHC and transitioning toward HVHS.

## Key Points

**Question:**

How have value-based health care (VBHC) initiatives been introduced and scaled up in health systems globally to improve health outcomes, reduce costs, and contribute to building high-value health systems?

**Findings:**

This scoping review of 50 initiatives found that the implementation of VBHC globally is still in its early stages, with published scientific literature pointing to small-scale institutional-level implementation within individual departments and hospitals. Large-scale implementation designed to develop high-value health systems is limited.

**Meaning:**

The narrow scope and scale of existing VBHC initiatives reinforce the need to develop comprehensive strategies that can drive systemwide implementation and adoption.

## Teaser

This scoping review examines how value-based health care initiatives have been adopted in health systems worldwide by analyzing their adoption, their characteristics, and their alignment with the value-based health care and high-value health systems frameworks.

## Introduction

Health systems globally face several contextual challenges, including slow economic growth leading to fiscal constraints, workforce shortages, aging populations with complex health care needs, and persistent health disparities.^1,2,3^ These challenges are leading to rising health care costs and increased demand for health services, posing a threat to health system sustainability.^4^ There is also a rise in citizen expectations for greater accountability to ensure financial protection while improving access to health services that are more effective and efficient, creating value for money, and services that are equitable and responsive to user expectations, thereby creating value for many.^5^ Redesign of health systems is necessary to modify governance and organization, financing, and resource management to produce appropriate outputs (public health and individual health services) that achieve improved health outcomes for individuals, communities, and society at large.

Value-based approaches to health care have been proposed as a way to improve patient-related outcomes and to reduce costs within health systems. The value-based health care (VBHC) framework was introduced in the US in 2006 to combat rising health care expenditures that failed to produce improvements in patient quality, safety, and outcomes over the past decades. The framework focuses on 6 elements: (1) organizing care around medical conditions, (2) measuring outcomes and costs for every patient, (3) aligning reimbursement with value through bundled payments, (4) integrating care systems regionally, (5) establishing national centers of excellence for complex care, and (6) using information technology systems to support these elements. VBHC is posited as a transformative strategy that catalyzes competition and creates greater value in health care by maximizing patient outcomes while minimizing associated costs.^6,7^ Specifically, value creation and delivery are centered around medical conditions and cycles of care while aiming to capture patient-reported outcomes and experiences.^8^ This framework represents a pivotal shift toward the delivery of care that prioritizes patients’ needs and related outcomes.

The high-value health systems (HVHS) framework identifies components that need to be in place to deliver at-scale public health and individual health services that are effective, efficient, equitable, and responsive to improve the health of individuals and populations, achieve financial protection, and ensure user satisfaction. The HVHS framework comprises 10 interdependent value-creating components: (1) digital data systems, (2) analytics, (3) cost-measurement systems, (4) outcome-measurement systems, (5) benchmarking, (6) integrated care pathways with bundled services, (7) value-based payment models, (8) value-based procurement, (9) integrated provider networks, and (10) strategic change and innovation ecosystems.^9^ Implementation of these components can help transform health systems to produce effective public health and individual health services that are delivered efficiently to achieve greater value for money, but in ways that are equitable and responsive to citizen expectations to achieve value for many.^5,10^

We conducted a scoping review of VBHC initiatives, defined as any program or intervention designed to improve value in health systems, implemented between 2007 and 2023. We defined value in the context of health care as any approach or action with the primary intent to improve health outcomes in terms of effectiveness, efficiency, equity, and responsiveness.

We examined how VBHC initiatives have been adopted in health systems worldwide, by analyzing (1) their adoption, (2) their characteristics, and (3) their alignment with the VBHC and HVHS frameworks. We use this analysis to explore potential pathways for health system leaders to scale VBHC initiatives.

## Methods

### Search Strategy

We adopted the updated Arksey and O’Malley framework as proposed by Peters et al,^11^ which describes scoping reviews as a method of mapping and examining current literature about a topic to identify gaps and inform current practice. To identify the relevant literature pertaining to VBHC initiatives in the broadest sense, we searched for studies discussing the framework in relation to health outcomes or quality of care and health care costs. We searched the following databases: MEDLINE, PubMed (National Library of Medicine), Embase (Elsevier), Health Business Elite (EBSCO), and Web of Science Core Collection (Clarivate). We included controlled vocabulary terms (ie, MeSH [Medical Subject Headings], Emtree, Health Business Elite subjects) when available and appropriate. The search strategies were designed and carried out by a health sciences librarian (C.M.), based on discussion with the team. The publication date of articles was from January 1, 2007, to July 7, 2023, and was applied to coincide with the introduction of the VBHC framework in 2006. Language limits were not applied. The exact search terms used in each of the databases, and corresponding result numbers, are provided in eAppendix 1 in Supplement 1. We adhered to the Preferred Reporting Items for Systematic Reviews and Meta-Analyses extension for Scoping Reviews (PRISMA-ScR) to ensure a comprehensive and systematic evaluation of the implementation strategies for initiatives.^12^

### Inclusion and Exclusion Criteria

We included empirical studies that evaluated the implementation of VBHC initiatives in health systems. Experimental studies, observational studies, and case studies that addressed the implementation of 1 or more of the components of the VBHC framework were included.^8^ Studies solely focused on health insurance, cost-effectiveness analysis, calculation of value for interventions in clinical settings, and design models or proposals without true implementation of value-based initiatives were excluded. Nonempirical studies and gray literature, such as review articles, commentaries, abstracts, book chapters, letters, news articles, conference papers, and proceedings, were also excluded.

### Screening, Data Extraction, and Analysis

Our screening process was carried out in 2 stages by independent reviewers, first conducting title and abstract screening, then progressing to full-text reviews. From the included studies, we extracted information on publication details, geographical data, contextual information on the health systems where value-based initiatives were implemented, and characteristics of the value-based initiative and its implementation. This structured approach allowed us to capture a breadth of insights into the initiatives, encompassing the challenges addressed, timing of implementation, adopters, and the settings in which these were deployed, ranging from national to departmental levels. We examined how each initiative aligned with the components of the HVHS framework to assess integration into health systems at scale (eAppendices 2 and 3 in Supplement 1).

## Results

### Search Findings

**Figure 1.**
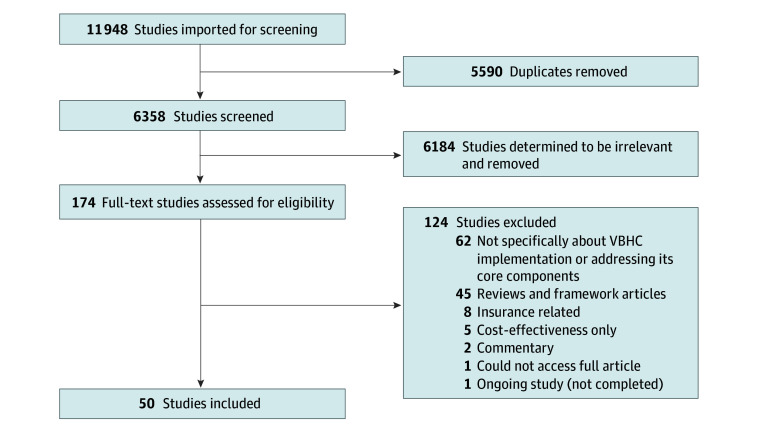
Flowchart of Study Selection VBHC indicates value-based health care.

The database search yielded 11 948 articles. After removing 5590 duplicates, 6358 unique articles underwent title and abstract screening, leading to the selection of 174 articles for full-text review. Fifty studies were included for data extraction, as illustrated in Figure 1.

### Characteristics of Included Initiatives

**Figure 2.**
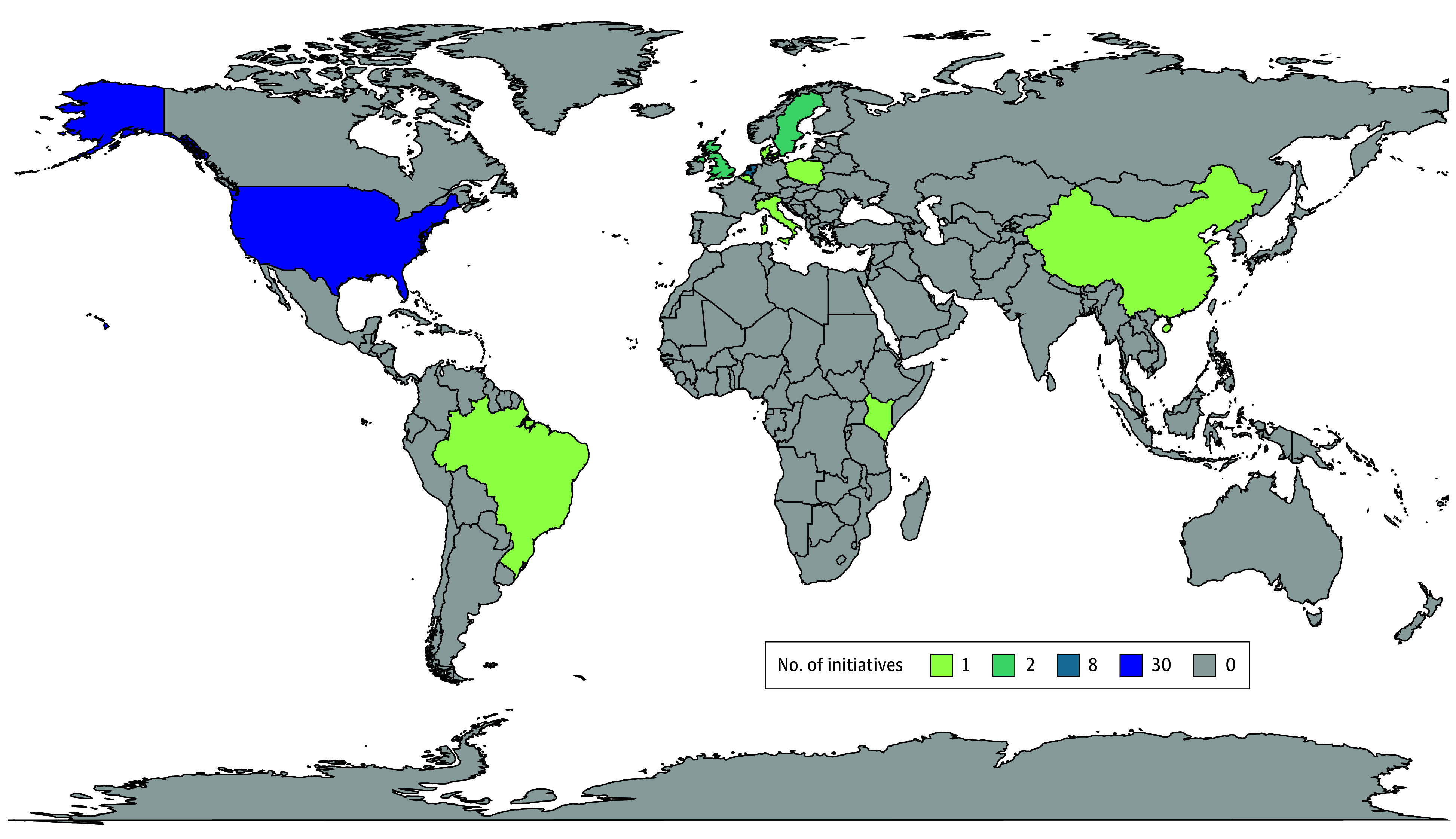
Geographic Distribution of Countries Featured in the Value-Based Health Care Initiatives

In this scoping review, 47 initiatives were from high-income countries, 2 were from upper-middle–income countries, and 1 initiative was from a lower-middle–income country. Within the represented countries, 31 initiatives originated from the US, 8 in the Netherlands, 2 in Sweden, 1 in Brazil, 2 in the UK, 1 in Italy, 1 in Belgium, 1 in Denmark, 1 in Poland, 1 in China, and 1 in Kenya. A geographic map with represented countries highlighted is shown in Figure 2.

**Figure 3.**
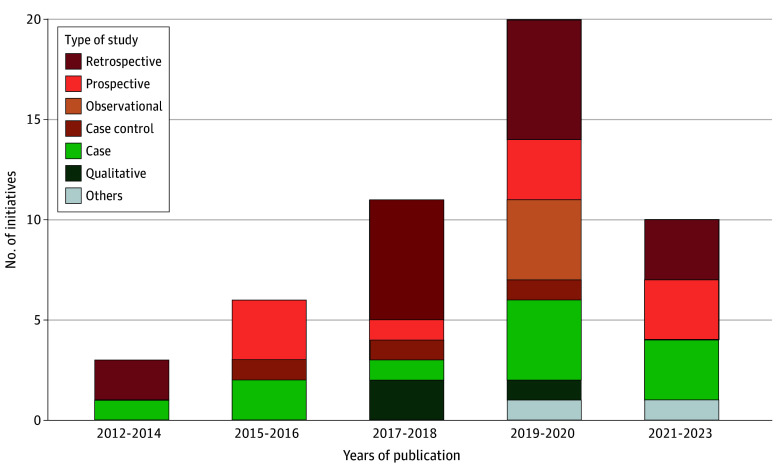
Study Characteristics of the Value-Based Health Care Initiatives

The study designs that evaluated or described the initiatives varied considerably between 2012 and 2023, including 17 retrospective studies, 10 prospective studies, 3 case-control studies, 11 case studies, 3 qualitative studies, 4 observational studies, 1 quasi-experimental study, and 1 mixed-methods study. Notably, most of these studies were published between 2017 and 2020, highlighting a growing focus on VBHC initiatives during this period (Figure 3).

### Challenges the Initiatives Were Designed to Address

Most studies described the need for VBHC initiatives in terms of the immediate challenges at the organizational level—in particular, the rise in volume of health care services required to manage certain conditions with variable health outcomes and uncontrolled costs. Motivations behind implementing VBHC initiatives included the ability to provide health care services equitably to the populations served, the need for more responsive care, and the requirement for health care professionals to understand the costs of the care they deliver.^13,14,15^

Often, these immediate challenges at the organizational level were also framed in terms of broader health system challenges, including rising national health care expenditures, variations or unknown outcomes in terms of quality of care provided, ineffective payment systems such as fee for service that focus on volume,^14,16,17,18,19,20,21^ fragmented delivery of care,^16,22,23,24,25,26,27,28^ health inequities,^20,29^ and the rapid demographic and epidemiologic shifts leading to an aging population and a rise in chronic conditions.^13,19,30,31,32^ A minority of studies reported a lack of focus on outcomes that matter to patients in health care decision-making.^15,33,34,35^

### Key Adopters of Initiatives

The VBHC initiatives examined were often supported by health care professionals within departmental units (eAppendix 4 in Supplement 1), prompted by an increasing awareness about VBHC, preparation for system transitions toward bundled payments, new legislation, and perceived health system problems such as fragmentation of care and suboptimal health outcomes against a background of increasing health care costs. Nine studies described top-down approaches to the implementation of value-based initiatives through hospital or health system administrative leaders.^18,26,28,36,37,38,39,40,41^ In the case of bundled payments, payers (often government insurance systems) were important adopters.^14,16,19,26,29,30,32,37,42^ Importantly, 4 studies reported patients as crucial stakeholders in the implementation of VBHC initiatives, specifically around the use of patient-reported outcome measures (PROMs).^15,25,34,43^

### Scope of Initiatives

#### Elements of the VBHC Framework Implemented

The VBHC elements included in each initiative varied considerably, but none included all 6 elements of the VBHC framework. In the initiatives reviewed, VBHC was introduced in different ways. Twenty-six initiatives introduced VBHC through integrated care pathways, including 9 through integrated practice units,^14,19,20,22,23,24,25,26,27,28,30,31,35,37,38,41,43,44,45,46,47,48,49,50,51,52^ and 16 VBHC initiatives introduced outcome measures, with 3 prioritizing primarily PROMs in the initial implementation.^15,17,18,21,25,28,30,33,34,35,36,38,39,41,47,52^ Most of the initiatives included cost measurements, and many focused on direct costs. Five initiatives used activity-based costing measures.^21,28,36,40,53^ Eleven initiatives described introducing the implementation of VBHC through bundled payments.^14,16,19,26,29,30,32,37,42,52,54^ This was particularly common in the US, with national reforms toward value-based purchasing by federal government agencies such as the Centers for Medicare & Medicaid Services. Five studies described the integration of care delivery across sites, which led to greater volume of care provided.^23,28,45,52,55^

Additionally, most settings had a technological platform such as electronic medical records that was used to track patient clinical outcomes. However, only 14 studies reported data analytics and/or benchmarking to compare outcomes and costs across departments, institutions, or regions.^17,21,23,25,30,33,36,37,38,43,45,47,52,56^

#### Components of the HVHS Framework Included in VBHC Initiatives

The VBHC initiatives studied incorporated several components of the HVHS framework. The predominant components across the studies included integrated care pathways with bundled services,^15,16,17,18,21,23,24,25,27,30,31,36,37,38,39,40,41,42,44,45,46,47,50,52,53,56,57,58,59^ outcome measurement systems,^14,15,16,17,18,19,20,22,23,24,25,26,27,28,29,30,31,32,33,34,35,36,37,38,39,40,41,42,43,44,45,46,47,48,49,50,51,52,56,57,59^ cost measurement systems,^15,16,17,18,19,20,21,22,23,24,25,26,28,29,31,32,33,36,37,38,39,40,42,43,44,45,46,47,48,49,50,53,57,59,60^ and performance benchmarking.^17,21,22,25,27,31,37,45,47,48,49,52,53,55,58,60,61^

The VBHC initiatives studied often involved redesigning service delivery processes to improve efficiency and patient outcomes, with a strong emphasis on integrated care pathways and outcome measurements. Strong governance and organizational support, including leadership changes and clear institutional priorities, were cited in some initiatives as being critical enablers of VBHC.^27,36,39^ Financial payment models, particularly bundled payments, often served as an impetus for the transition to a focus on value rather than volume.^14,16,19,26,30,32,37,42,54^ The use of electronic health records and digital platforms was seen consistently across studies.^13,14,15,17,18,19,21,22,23,25,27,30,33,36,37,38,41,42,44,45,47,49,50,52,54,55,57,61^

### Scale of Initiatives: Level of Implementation Within the Health System

When looking at the implementation of initiatives within health systems, the scale varied, with many initiatives rooted in specific departments and hospitals. Twenty-two initiatives described implementation in individual clinical departments.^14,15,16,17,20,23,24,25,29,31,33,34,38,41,42,43,44,45,46,50,53,56^ Nine initiatives described implementation across different hospital departments.^19,26,28,30,35,39,47,48^ Eight initiatives alluded to specific multihospital or organization networks.^21,22,27,36,37,40,49,54^ Larger-scale diffusion of initiatives across health systems was limited, with 8 studies describing regional initiatives^13,18,32,52,57,58,60,62^ and 4 initiatives suggesting national-level implementation.^51,55,59,61^

**Figure 4.**
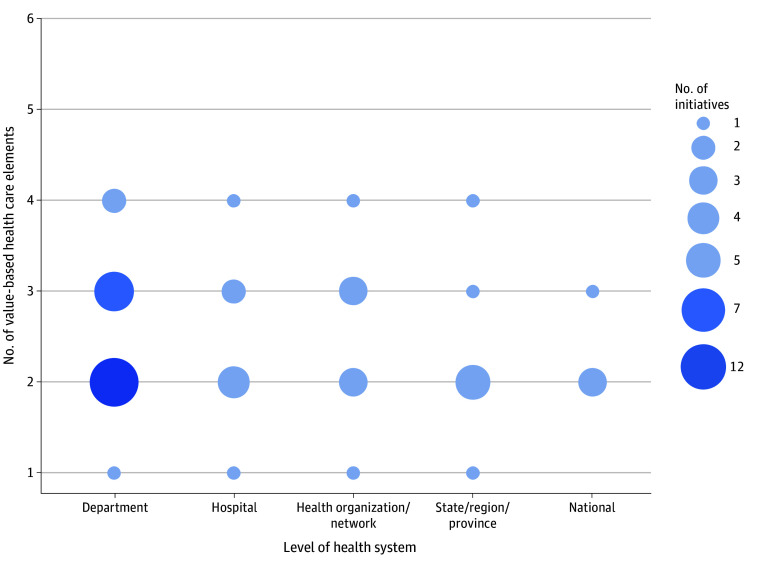
Adoption of Value-Based Health Care Elements Across Health System Levels and Number of Value-Based Health Care Elements per Initiative

**Figure 5.**
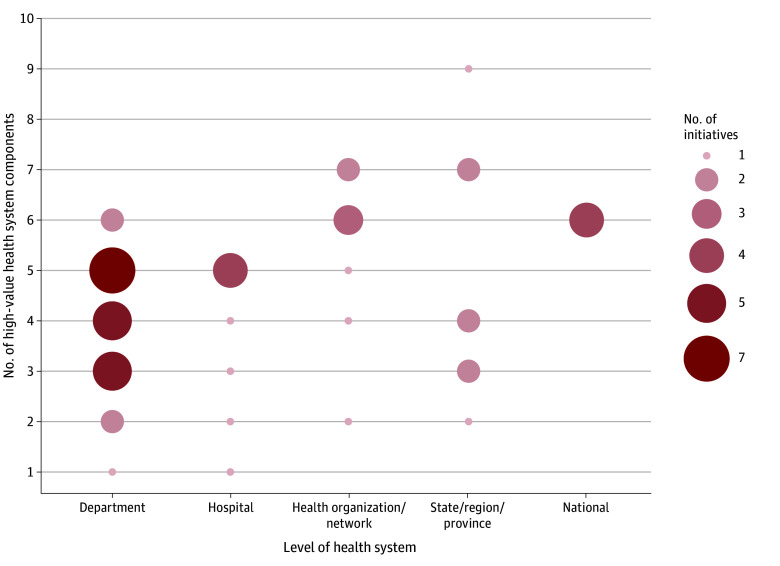
Number of High-Value Health System Components Across Health System Levels

When comparing the number of VBHC elements and HVHS components within each initiative, there is a similar trend. Most of the initiatives analyzed contained 2 to 3 VBHC elements (Figure 4) and 3 to 5 HVHS components (Figure 5). Notably, no initiative included all 6 VBHC elements or the 10 HVHS components. However, initiatives that had a higher number of HVHS components tended to aggregate at higher levels within the health system (ie, health organization/network level and above; Figures 4 and 5).^18,51,52,59^

## Discussion

This review critically examines the adoption and diffusion of VBHC initiatives in health systems globally, as well as their alignment with the VBHC and HVHS frameworks. The study identified that VBHC initiatives are at an early adoption stage across health systems. Of the 50 initiatives identified, 47 were from high-income countries (31 [66%] of which were from the US), 2 from upper-middle–income countries, and 1 from a lower-middle–income country.

Transitioning from a volume- to a value-based payment model represents a considerable shift in how health systems operate.^8^ Such a transition requires major strategic change. Thus, it is not surprising that for many of the initiatives examined, implementation is often confined to departmental levels. For example, in cardiac rehabilitation programs, particularly in the US, integrated care pathways have been developed to optimize patient outcomes while managing costs.^38^ However, these efforts have not yet been coordinated at national or regional levels. Nonetheless, some European VBHC initiatives for cardiovascular care have achieved national scale. For instance, the Netherlands Heart Registry coordinates a nationwide initiative that collects outcomes and costs for patients with cardiac disease, which enables benchmarking of best practices and helps to inform quality improvement.^55^ These differences in implementation scope may be explained by the centralized financing structure of European health care systems compared to the largely decentralized, multipayer insurance-based system in the US.

While a few elements of the VBHC framework (ie, measuring costs and outcomes) are being implemented at the national level, the practical application of this information—through reimbursements and organizing care—remains primarily at the departmental level within health care institutions. For instance, in the US, national VBHC initiatives like the Bundled Payments for Care Improvement and the Comprehensive Care for Joint Replacement programs are designed for specific disease clusters, particularly in orthopedics due to the high volume and cost of procedures.^19,37^ Despite a national goal for payment reforms to transition from volume- to value-based care, these programs are still primarily executed at the departmental and/or hospital level.^45,46^ This may be, in part, due to the structure of financing that promotes budgetary boundaries between departments.^39^ Overall, the studies did not reveal coordinated national approaches to fully shift from volume to value. This slow adoption reinforces the need for an enabling ecosystem that will help catalyze integrated systemwide transformation.

Additionally, while most represented VBHC initiatives reported using digital platforms through electronic medical records to measure patient outcomes and costs, there were only a few reports of the use of digital data for real-time analytics with performance benchmarking of costs and outcomes for care episodes. For system changes at the population level, benchmarking would be critical for identifying gaps and driving motivation for improvement toward best practices in care at all levels.

The HVHS framework, informed by studies focusing on the G20 countries, extends the VBHC framework by proposing 10 components that need to be in place to gradually transition to a HVHS that provides effective, efficient, equitable, and responsive health services to achieve improved population health outcomes, financial protection, and user satisfaction at scale.^10^ Several countries are implementing components of HVHS at the national level. For example, the National Health Service in England, the Canadian Institute for Health Information, and the Netherlands Heart Registry have implemented digital data systems with analytics that track costs and outcomes, including PROMs, to track performance over time.^10,55,63^

Canada has been implementing elements of HVHS at the provincial and national levels. A notable example is the shift toward value-based procurement for medical devices—a component that was not explicitly mentioned in any of the VBHC initiatives analyzed. A value-based procurement approach ensures that purchasing decisions are based not just on cost, but also on the value medical devices bring to patients and institutions.^64^ In addition, the Pan-Canadian Health Data Strategy has been established to enhance data use in improving patient outcomes and the efficiency of provincial health systems.^65^ Condition-specific PROMs for joint replacement surgeries are being collected, alongside the national efforts to standardize the collection of generic PROMs.^63^

Overall, this study found limited published data about VBHC implementation in lower-middle–income countries. This is likely due to several challenges related to VBHC design, including financing. Nevertheless, a study based in Kenya has shown that with the adaptation of local and cultural contexts, implementation of value-based care delivery is possible.^52^ Using incremental cohort-based implementation of maternal bundled care, digital platforms using integrated mobile money and text-based communications were highlighted as a key to implementation success. This finding reinforces the role of technology in HVHS design and the need for context-specific, tailored implementation strategies, particularly in lower-resource settings.

### Limitations

The study has several limitations. First, although we developed a university library protocol for scoping reviews and adhered to PRISMA guidelines, the protocol was not registered, potentially affecting the study’s replicability, although the search details are provided in eAppendix 1 in Supplement 1. Second, the articles included in this review were predominantly written in English language, and studies in other languages may have been missed. Third, while this review primarily focuses on the introduction of VBHC initiatives rather than their long-term outcomes, identifying the barriers to upstream implementation at this early stage is crucial for enabling future efforts. Although some observational studies from the US present a mixed picture,^66^ the present findings highlight the necessity for continued longitudinal research to assess the true impact of VBHC initiatives within health systems.

## Conclusions

This scoping review examined the global implementation of VBHC initiatives and their alignment with the VBHC and HVHS frameworks. Currently, VBHC initiatives are often confined to small scales, such as individual departments and hospitals, and are less frequently integrated into national health systems. The HVHS framework offers a pathway to amplify these initiatives through 10 distinct components that can help improve the effectiveness, efficiency, equity, and responsiveness of health services produced by health systems to achieve greater value. This study shows that most VBHC initiatives implemented were in high-income countries, with a relative absence of studies available in lower-middle–income countries. The findings highlight the need for better evidence on what works in different settings for VBHC implementation, as well as the long-term benefits of various approaches to address health system challenges unique to each country’s context.

## Notes

Supplement. **eAppendix 1.** Search Terms **eAppendix 2.** Key Components of the High-Value Health System Model **eAppendix 3.** Definitions of the 10 Component of the High-Value Health System Model **eAppendix 4.** Definitions of Unit of Implementation

Supplement 2.
Data Sharing Statement
