# Supplementary material for: Global Adoption of Value-Based Health Care Initiatives Within Health Systems: A Scoping Review
Source: JAMA Health Forum. 2025 May 16;6(5):e250746. doi: 10.1001/jamahealthforum.2025.0746 (PMC12084849; doi:10.1001/jamahealthforum.2025.0746)
Supplement: Supplement. — eAppendix 1. Search Terms eAppendix 2. Key Components of the High-Value Health System Model eAppendix 3. Definitions of the 10 Component of the High-Value Health System Model eAppendix 4. Definitions of Unit of Implementation [file jamahealthforum-e250746-s001.pdf]

Supplemental Online Content

Douglas AO, Senkaiahliyan S, Bulstra CA, Mita C, Reddy CL, Atun R. Global adoption of value-based health care initiatives within health systems: a scoping review. *JAMA Health Forum*. 2025;6(5):e250746. doi:10.1001/jamahealthforum.2025.0746

eAppendix 1. Search Terms
eAppendix 2. Key Components of the High-Value Health System Model
eAppendix 3. Definitions of the 10 Components of the High-Value Health System Model
eAppendix 4. Definitions of Unit of Implementation

This supplemental material has been provided by the authors to give readers additional information about their work.

© 2025 Douglas AO et al. *JAMA Health Forum*.

eAppendix 1. Search Terms

Medline / PubMed (National Library of Medicine, NCBI)

4,736 records 7 July 2023

("Value-Based Purchasing"[Mesh] OR "Value-Based Health Insurance"[Mesh] OR value based care[tiab] OR value based delivery[tiab] OR value based health care[tiab] OR value based healthcare[tiab] OR value based purchasing[tiab] OR value driven care[tiab] OR value driven health care[tiab] OR value driven healthcare[tiab])
OR
((value based[tiab] OR value driven[tiab])
AND
("Insurance, Health"[Mesh:NoExp] OR "Insurance, Health, Reimbursement"[Mesh] OR insurance[tiab] OR reimbursement*[tiab] OR payment*[tiab]))
OR
((value based[tiab] OR value driven[tiab])
AND
("Treatment Outcome"[Mesh] OR "Outcome Assessment, Health Care"[Mesh:NoExp] OR "Patient Outcome Assessment"[Mesh:NoExp] OR "Patient Reported Outcome Measures"[Mesh:NoExp] OR outcome*[tiab] OR "Quality of Health Care"[Mesh:NoExp] OR "Quality Improvement"[Mesh:NoExp] OR "Quality Indicators, Health Care"[Mesh:NoExp] OR "Quality Assurance, Health Care"[Mesh:NoExp] OR quality[tiab])
AND
("Health Care Costs"[Mesh] OR "Cost Control"[Mesh] OR cost[tiab] OR costs[tiab] OR costing[tiab]))
AND
2007[pdat]:2023[pdat]
NOT
("Editorial"[ptyp] OR "Comment"[ptyp] OR "Letter"[ptyp] OR "News"[ptyp])

Embase (Elsevier, embase.com)

3,582 records 7 July 2023

Advanced Search
Remove Embase mapping options
Date: Publication Years from: 2007 – 2023
Source: Embase (1974 to present)

1
('value-based insurance design'/de OR 'value based care'/de OR 'value based health care'/de OR 'value based payment'/de) NOT ('conference abstract'/it OR 'conference paper'/it OR 'editorial'/it OR 'letter'/it)

2)
('value based care' OR 'value based health care' OR 'value based delivery' OR 'value based healthcare' OR 'value based purchasing' OR 'value driven care' OR 'value driven health care'

© 2025 Douglas AO et al. *JAMA Health Forum*.

OR 'value driven healthcare'):ab,ti,kw NOT ('conference abstract'/it OR 'conference paper'/it OR 'editorial'/it OR 'letter'/it)

3)
('value based' OR 'value driven'):ab,ti,kw AND ('health insurance'/de OR 'reimbursement'/de OR (insurance OR reimbursement* OR payment*):ab,ti,kw) NOT ('conference abstract'/it OR 'conference paper'/it OR 'editorial'/it OR 'letter'/it)

4)
('value based' OR 'value driven'):ab,ti,kw AND ('treatment outcome'/exp OR outcome:ab,ti,kw OR 'health care quality'/de OR 'quality improvement study'/de OR quality:ab,ti,kw) AND ('health care cost'/exp OR 'cost control'/de OR cost*:ab,ti,kw) NOT ('conference abstract'/it OR 'conference paper'/it OR 'editorial'/it OR 'letter'/it)

1 OR 2 OR 3 OR 4

Health Business Elite (EBSCOhost)

398 records 7 July 2023

Remove: Apply equivalent subjects
Published date: 2007 – 2023
Limit results:
Peer Reviewed
Source Types: Academic Journals

1)
DE "VALUE-based healthcare"

OR
TITLE OR ABSTRACT:

"value based care" OR "value based health care" OR "value based delivery" OR "value based healthcare" OR "value based purchasing" OR "value driven care" OR "value driven health care" OR "value driven healthcare"

2)
TITLE OR ABSTRACT:
"value based" OR "value driven"

3)
DE "HEALTH insurance" OR DE "HEALTH insurance reimbursement" OR DE "BUNDLED payments (Medical care costs)"

OR
TITLE OR ABSTRACT:
insurance OR reimbursement* OR payment*

© 2025 Douglas AO et al. *JAMA Health Forum*.

4)
DE "TREATMENT effectiveness" OR DE "HEALTH outcome assessment" OR DE "PATIENT reported outcome measures" OR DE "MEDICAL quality control" OR DE "QUALITY assurance"

OR
TITLE OR ABSTRACT:
outcome* OR quality

5)
DE "MEDICAL care costs" OR DE "MEDICAL care cost control"

OR
TITLE OR ABSTRACT:
cost*

1 OR (2 AND 3) OR (2 AND 4 AND 5)

Web of Science Core Collection (Clarivate)

3,232 records 7 July 2023

Include editions:
Science Citation Index Expanded
Social Sciences Citation Index

Advanced Search:
More options: Exact search
Date Range: Custom 2007-01-01 to 2023-07-07
Exclude – Document Types: Editorial Materials, Meeting Abstracts, Proceedings Papers, Letters, News Items, Book Chapters

1)
TS=("value based care" OR "value based health care" OR "value based delivery" OR "value based healthcare" OR "value based purchasing" OR "value driven care" OR "value driven health care" OR "value driven healthcare")

2)
TS=(("value based" OR "value driven") AND (insurance OR reimbursement* OR payment*))

3)
TS=(("value based" OR "value driven") AND (quality OR outcome*) AND cost*)

#1 OR #2 OR #3

© 2025 Douglas AO et al. *JAMA Health Forum*.

eAppendix 2. Key Components of the High-Value Health System Model. Source:Health Systems Innovation Lab, Harvard University, 2024; reprinted with permission.

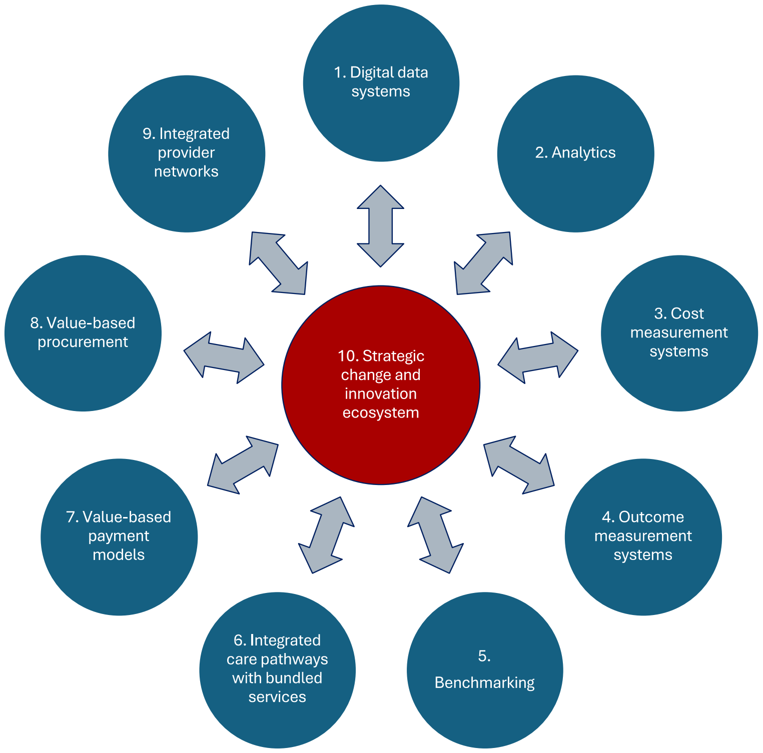

© 2025 Douglas AO et al. *JAMA Health Forum*.

eAppendix 3. Definitions of the 10 Components of the High-Value Health System Model

**Digital Data Systems**	Platforms that enable the reporting, collection, and pooling of relevant healthcare data for all major actors within the system.
**Analytics**	The use of digital data systems to deploy advanced data science capabilities on relevant health data to enhance healthcare service delivery and policy development.
**Cost Measurement Systems**	Harmonizing cost definition and measurement to enable value-based payment and comparative benchmarking of healthcare costs.
**Outcome Measurement Systems**	Development of harmonized indicators, measurement, and comparative benchmarking of outcomes related to health service delivery and patient experiences.
**Performance Benchmarking**	Collection and analysis of data enabling comparison over time or across units to improve health system performance.
**Integrated Care Pathways with Bundled Services**	Standardization of processes for healthcare services and bundling interventions to promote efficiency and improve outcomes.
**Value-Based Payment Models**	Implementation of payment models where healthcare professional payments are based on improving outcomes and reducing the cost of care delivered.
**Value-Based Procurement**	Shifting from purchasing inputs to paying for outcomes, with risk/reward alignment and supply chain optimization to improve efficiency in procurement processes.
**Integrated Provider Networks**	Operational and structural integration of healthcare services to enable seamless care across pathways and improve efficiency and outcomes.
**Strategic Change and Innovation Ecosystem**	Leadership and institutionalization of policies that promote innovation design, development, implementation, and scale-up to transform health systems for value creation.

© 2025 Douglas AO et al. *JAMA Health Forum*.

eAppendix 4. Definitions of Unit of Implementation.

Unit of Implementation	Definition
**Department**	A specific division within a hospital or health organization responsible for particular functions or services.
**Hospital**	A single health facility providing medical and surgical treatment and nursing care for sick or injured people.
**Health Organization/Network**	A group of healthcare professionals or facilities that work together to deliver coordinated care across multiple settings.
**State/Region/Province**	A subnational administrative division within a country that has jurisdiction over health policy and services.
**National**	The entire country's health system, encompassing all levels of healthcare delivery and policy implementation.

© 2025 Douglas AO et al. *JAMA Health Forum*.
