# Supplementary material for: Global Adoption of Value-Based Health Care Initiatives Within Health Systems: A Scoping Review
Source: JAMA Health Forum. 2025 May 16;6(5):e250746. doi: 10.1001/jamahealthforum.2025.0746 (PMC12084849; doi:10.1001/jamahealthforum.2025.0746)
Supplement: Supplement 2. — Data Sharing Statement [file jamahealthforum-e250746-s002.pdf]

Data Sharing Statement

Douglas. Global Adoption of Value-Based Health Care Initiatives Within Health Systems. *JAMA Health Forum*. Published May 16, 2025. doi:10.1001/jamahealthforum.2025.0746

Data
Data available: Yes
Data types: Data (not involving human participants)
How to access data: With the publication in the Methods, Results, Figures, Supplementary File, and References.
When available: With publication

Supporting Documents
Document types: Other (please specify)
Additional Information: Search strategy
How to access documents: Supplementary file Data extraction sheet available upon reasonable request (ayooluwa_douglas@hms.harvard.edu)
When available: With publication

Additional Information
Who can access the data: Anyone requesting the data
Types of analyses: For any purpose
Mechanisms of data availability: With investigator support
